# A Truncating Variant in the *ERCC6* Gene With Three Different Phenotypes: Significant Effects of Modifier Genes

**DOI:** 10.1155/genr/7396691

**Published:** 2025-12-25

**Authors:** Mehdi Khorrami, Erfan Khorram, Mohammad Amin Tabatabaiefar, Omid Yaghini, Omid Iravani, Aida Kheirollahi, Majid Kheirollahi, Vida Yazdani, Mitra Pakbaz

**Affiliations:** ^1^ Department of Genetics and Molecular Biology, School of Medicine, Isfahan University of Medical Sciences, Isfahan, Iran, mui.ac.ir; ^2^ Applied Physiology Research Center, Cardiovascular Research Institute, Isfahan University of Medical Sciences, Isfahan, Iran, mui.ac.ir; ^3^ Cancer Prevention Research Center, Seyyed Al-Shohada Hospital, Isfahan University of Medical Sciences, Isfahan, Iran, mui.ac.ir; ^4^ Research Institute for Primordial Prevention of Non-Communicable Disease, Child Growth and Development Research Center, Isfahan University of Medical Sciences, Isfahan, Iran, mui.ac.ir; ^5^ Department of Child Neurology, Isfahan University of Medical Sciences, Isfahan, Iran, mui.ac.ir; ^6^ Legal Medicine Research Center, Legal Medicine Organization, Tehran, Iran, lmo.ir; ^7^ Geneazma Medical Genetics Laboratory, West Shariati St., Isfahan, Iran; ^8^ Department of Biology, Islamic Azad University, Falavarjan Branch, Isfahan, Iran, srbiau.ac.ir; ^9^ Department of Biology, Islamic Azad University East Tehran Branch, Tehran, Iran, srbiau.ac.ir; ^10^ Islamic Azad University, Flavarjan Branch, Flavarjan, Isfahan, Iran, srbiau.ac.ir

**Keywords:** cerebrooculofacioskeletal syndrome (COFS), Cockayne syndrome, *ERCC6*, intermediate phenotype, modifier genes

## Abstract

**Background:**

Cockayne syndrome (CS) is a rare, autosomal‐recessive, multisystem disorder characterized by microcephaly, failure to thrive, photosensitivity, leukodystrophy, muscle contracture, and intellectual disability. It is caused by deleterious variant in the *ERCC6* and *ERCC8* genes, which are involved in the transcription‐coupled nucleotide excision repair system. According to severity and age of onset, CS is categorized into four types: I, II, III, and cerebrooculofacioskeletal syndrome (COFS). However, some researchers consider COFS to be a distinct disease from CS, while others describe COFS as a severe form of CS.

**Methods:**

Whole‐exome sequencing (WES) and Sanger sequencing were used to identify potential pathogenic causative variant.

**Results:**

WES data analysis revealed a nonsense variant (NM_000124: c.3862C>T, p.R1288X) in the *ERCC6* gene, which was co‐segregated using Sanger sequencing. Although this variant has been reported previously in association with both CS and COFS separately, this study’s patient manifested intermediate symptoms.

**Conclusion:**

This study’s findings expand the clinical spectrum of the variant (NM_000124: c.3862C>T, p.R1288X) and provide more supporting evidence that CS and COFS are phenotypic spectrums rather than different clinical conditions in which genetic and epigenetic factors probably play a pivotal role in the severity of symptoms.

## 1. Introduction

Cockayne syndrome (CS) is a rare autosomal recessive multisystem disorder caused by a deficiency in nucleotide excision repair (NER), which was first described in 1936 by Sir Edward Cockayne [1]. It is a progressive disease characterized by failure to thrive, photosensitivity, leukodystrophy, muscle contracture, intellectual disability, loss of subcutaneous fat, recognizable facial appearance with deep sunken eyes, sensorineural hearing loss, and vision problems. Furthermore, in patients with CS, microcephaly usually develops within the first 2 years of life [2, 3]. Nance and Berry reviewed 140 patients with CS and provided detailed information on many of the clinical features. According to the age of onset and severity of symptoms, they proposed a three‐tiered classification system for CS, along with major and minor diagnostic criteria [4]. In CS type I, also known as the classic form, major disease characteristics become apparent by the age of one to 2 years. CS type II is a more severe form, in which abnormalities are recognized at birth or in the early neonatal period. Patients with CS type III showed later‐onset forms with a milder phenotype, in which its major features appear solely after the age of 2 years. The mean age of death in types I, II, and III is 16.1, 5.0, and 30.3, respectively [5, 6]. Molecularly, there are two complementary groups of CS: type A (CS‐A) and type B (CS‐B), which are caused by deleterious variant in the *ERCC8* and *ERCC6* genes, respectively [7, 8]. The *ERCC6* gene encodes a multifunctional protein called Cockayne syndrome B (CSB) that belongs to the SWI2/SNF2 family, which is involved in damaged DNA repair, chromatin remodeling, and transcription [9]. The CSB protein plays a crucial role in transcription‐coupled nucleotide excision repair (TC‐NER), which is responsible for repairing DNA lesions in the transcribed strand of active genes [3]. Moreover, this protein accumulates in a transcription‐dependent manner at sites of DNA double‐strand breaks (DSB) and regulates DNA DSB by activating homologous recombination (HR) and suppressing nonhomologous end joining (NHEJ) [10]. In addition to DNA‐repairing activities, CSB protein plays an essential role in the differentiation of the human neural progenitor cell, so at least some of the neurological symptoms observed in patients are due to CSB deficiency [11]. Several studies showed different CS types are a continuous spectrum, and there is no clear threshold among these types [12, 13]. Also, some study considers cerebrooculofacioskeletal syndrome (COFS) as a very severe fetal CS form with prenatal microcephaly, prenatal growth failure, arthrogryposis, and congenital cataracts, or microphthalmia, but others consider it as a distinct syndrome [14, 15].

This study examined a consanguineous family with two patients diagnosed with CS according to MRI and initial clinical findings. Whole‐exome sequencing (WES) revealed that the patients harbored a pathogenic variant (NM_000124: c.3862C>T, p.R1288X), which has previously been reported in association with COFS and CS, but detailed clinical evaluation revealed that our patient manifests an intermediate phenotype [13, 16]. Our study provides more evidence that the COFS and CS are spectra rather than different syndromes and unknown genetic or epigenetic factors play a vital role in the severity of the symptoms.

## 2. Materials and Methods

### 2.1. Subjects and Phenotype Investigation

**Figure 1:**
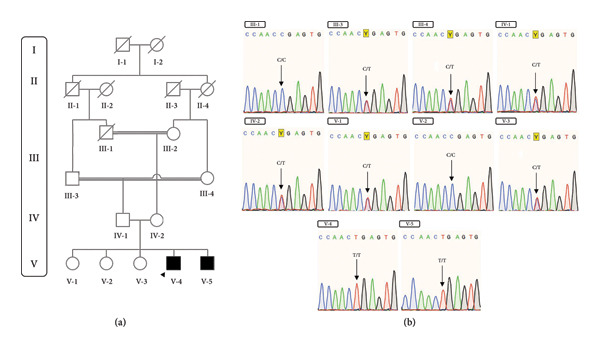
Pedigree of the studied family and the detected variant. (a) Pedigree chart of the family. The parents are double first cousins. (b) The results of the co‐segregation study are consistent with an autosomal recessive inheritance pattern of the variant in the *ERCC6* gene. Although the affected (V‐4, V‐5) members are homozygous for the (c.3862C>T, p.R1288X) deleterious variant, (III‐1, V‐2) and (III‐3, III‐4, IV‐1, IV‐2, V‐1, V‐3) which are healthy members of the family are wild‐type homozygous and heterozygous, respectively.

A consanguineous Iranian family with two affected individuals with growth failure and impaired developmental milestones was this study’s subject (Figure 1(a)). Written informed consent was obtained from all participants or their legal guardians. This study was carried out in accordance with the International Ethical Guidelines and Declaration of Helsinki and was approved by the Ethical Committee of Isfahan University of Medical Sciences (IR.MUI.MED.REC.1398.186). A detailed questionnaire was completed about family history, environmental risks, maternal complications during pregnancy, health history, developmental milestones, and disease progression.

### 2.2. Genomic DNA Extraction

Blood samples (5 mL) were collected in ethylenediaminetetraacetic acid (EDTA)–containing tubes from the healthy family members (father and mother) and affected siblings (Figure 1(a)). High‐quality genomic DNA was extracted using Exgene Blood SV mini kit (GeneAll Biotechnology Co, Ltd, South Korea) according to the manufacturer’s instructions. The quality and quantity of the extracted DNA were determined by agarose gel electrophoresis and Nanodrop 2000 spectrophotometer (Nanodrop 2000, Thermo Fisher Scientific, USA).

### 2.3. WES and Pathogenicity Assessment

The V‐4 (Figure 1(a)) patient DNA sample was sent to Macrogen (South Korea) (https://www.macrogen.com/) for WES. The library preparation and paired‐end DNA sequencing were performed using SureSelect Human All Exon V7 (Agilent Technologies, Inc., Santa Clara, CA, USA) and Novaseq 4000 platform (Illumina, San Diego, CA, USA) with coverage of 100 × mean depth, respectively. Briefly, the variant analysis was done via mapping the FASTQ to the reference genome (UCSC hg19) using the Burrows‐Wheeler Alignment software (https://bio-bwa.sourceforge.net/) with default parameters. Variants were called with Genome Analysis Tool Kit software (https://gatk.broadinstitute.org/) and annotated using ANNOVAR software. Variants with a minor allele frequency (MAF) < 0.01 were filtered in databases such as Exome Aggregation Consortium (ExAC) (https://exac.broadinstitute.org), the Genome Aggregation Database (gnomAD) (https://gnomad.broadinstitute.org/), 1000 Genomes Project Phase 3 Database (https://www.internationalgenome.org/), dbSNP Version 147, HGMD (https://www.hgmd.cf.ac.uk/ac/index.php), Exome Sequencing Project (ESP) (https://evs.gs.washington.edu/), and Iranome (https://www.iranome.ir/) to downstream analysis according to their chromosomal location, autosomal recessive mode of inheritance, functional consequences, and clinical presentation. Computational predictive tools such as MutationTaster (https://www.mutationtaster.org/), PROVEAN (https://provean.jcvi.org/index.php), SIFT Indel (https://sift-dna.org/), DDIG Indel (https://sparks-lab.org/ddig/), and PANTHER (https://www.pantherdb.org/) were used to predict the pathogenicity of the detected variant [17]. Interpretation of the results was performed according to the American College of Medical Genetics and Genomics (ACMG) guidelines [18].

### 2.4. Co‐Segregation Analysis via Sanger Sequencing

In order to confirm the candidate variant, specific forward (F) and reverse (R) primers including F: 5′‐CACATTGCTTGGGTTTATTTCC‐3′ and R: 5′‐TTCTAGCCTTAGTTGTTGGA‐3′ were designed in the flanking region of the identified variant. The PCR product was subsequently visualized using 1% agarose gel and bidirectional sequencing was carried out by an ABI 3130 sequencer (Applied Biosystems, USA). The result was compared with the *ERCC6* gene reference sequence (NG_009442.1) using SeqMan Ultra 17.2 software (https://www.dnastar.com/software/lasergene/seqman-ultra/).

## 3. Results

### 3.1. Clinical Description

**Figure 3:**
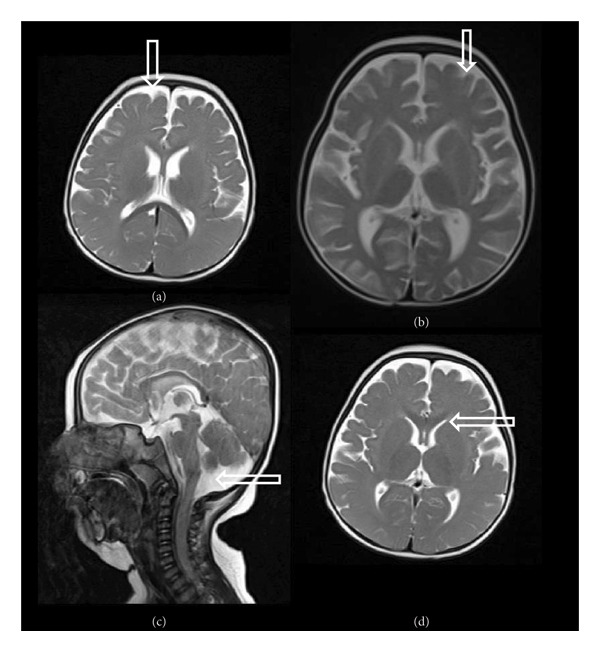
MRI study of proband. (a, b) Baseline magnetic resonance imaging examination performed at 3 years in subject V‐4 revealed brain atrophy with primary Sylvian fissure secondary to frontotemporal atrophy. (c) Increased size of retrocerebellar cisterna. (d) Diffuse symmetrical increase of signal intensity on periventricular white matter and subcortical white matter and corpus callosum thinning.

A consanguineous Iranian family with two affected individuals was the subject of this study (Figure 1(a)). Two of five (V‐4 and V‐5, Figure 1(a)) siblings, the product of a consanguineous marriage, presented with impaired developmental milestones, growth failure, and several neurologic symptoms. Parents and three older siblings were healthy with no important clinical conditions. During pregnancy, both affected siblings had intrauterine growth retardation, but their deliveries were term and uneventful. The proband (V‐4) was a three‐year‐old boy (Figure 2(a)). His birth weight and head circumference were 2.8 kg and 31.5 cm, respectively, and after birth, he was hospitalized for 3 days due to pulmonary insufficiency. Feeding problems and constipation were noted in the first month. Developmental milestones were normal until 4 months, and the subject almost had good head control, but after that, he could not reach the anticipated motor milestones. By the age of 2 years, the subject was able to sit with the help of occupational therapy, after which an arrest and regression happened. He showed anhidrosis, dry skin, loss of subcutaneous fat, and dermal photosensitivity and experienced facial erythema and perioral blistering after a period of exposure to sunlight, but there was no evidence of a permanent pigmentary (Figure 3). Now, at the age of three, head control is poor, and the subject cannot sit without support. Furthermore, he manifested intellectual disability, dysarthria, microcephaly, joint contracture, muscle weakness (more prominent in lower limbs than upper limbs), increasing difficulty in swallowing, dental caries, pulmonary insufficiency, and cold extremities. The ophthalmologic assessment revealed iris hypoplasia, strabismus, increased eye discharge, and optic atrophy. Audiological examinations showed bilateral mild and moderate hearing loss in the right and left ears, respectively. The facial features included deep‐set eyes, wide nasal bridge, abnormal nasal morphology, and a bird‐like face (Figures 2(b) and 2(c)). In physical assessment, pes planus, transverse palmar crease, growth retardation, short neck, scoliosis, and thin and sparse hair have been observed. Biochemistry analysis revealed proteinuria, elevated triglycerides, Serum Glutamic‐Pyruvic Transaminase (298 U/L), and serum glutamic oxaloacetic transaminase (157 U/L). His EEG was normal, and he had never experienced a seizure. MRI study at the age of 3 revealed brain atrophy with primary Sylvian fissure secondary to frontotemporal atrophy and increased size of retrocerebellar cisterna. Furthermore, diffuse symmetrical increase of signal intensity on periventricular white matter and subcortical white matter and corpus callosum thinning were seen (Figure 3). The second affected member in this family (V‐5, Figure 1(a)) was the younger brother of the proband. The birth weight and head circumference were 3 kg and 31 cm, respectively. At 6‐months‐old, he showed pulmonary insufficiency, feeding problems, mild to moderate hearing loss, developmental delay, and clinical cutaneous photosensitivity, which were similar to those of his affected brother.

Figure 2The pictures of the patient V‐4 at the age of 3 years. (a) V‐4 experienced facial erythema and perioral blistering after a period of exposure to sunlight, but there was no evidence of permanent pigmentary. (b, c) The facial features included deep set eyes, micrognathia, wide nasal bridge, abnormal nasal morphology, and bird‐like face. Also, he had thin and sparse hair.

**Figure:**
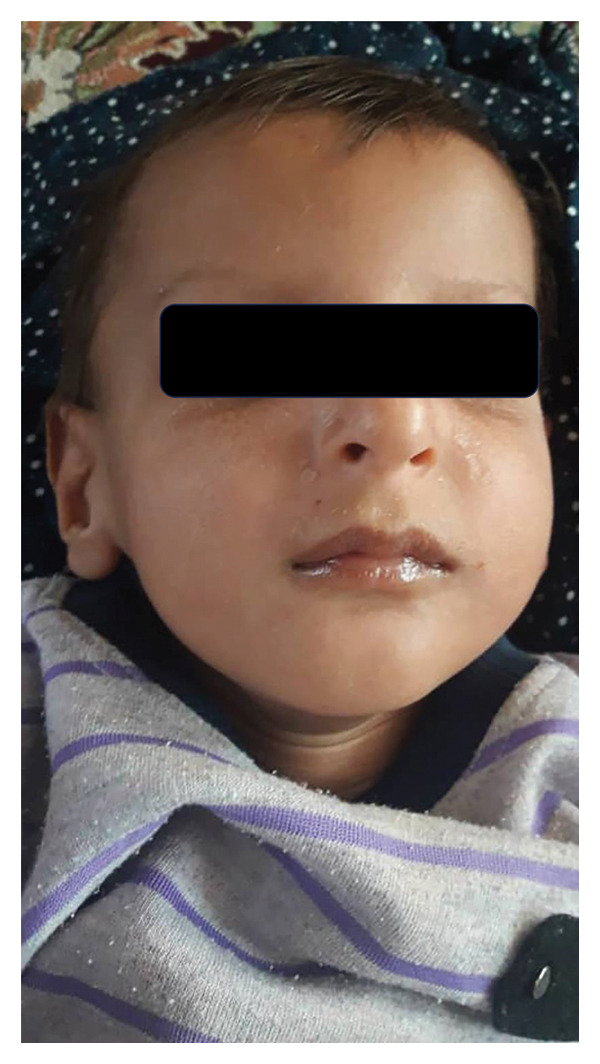
(a)

**Figure:**
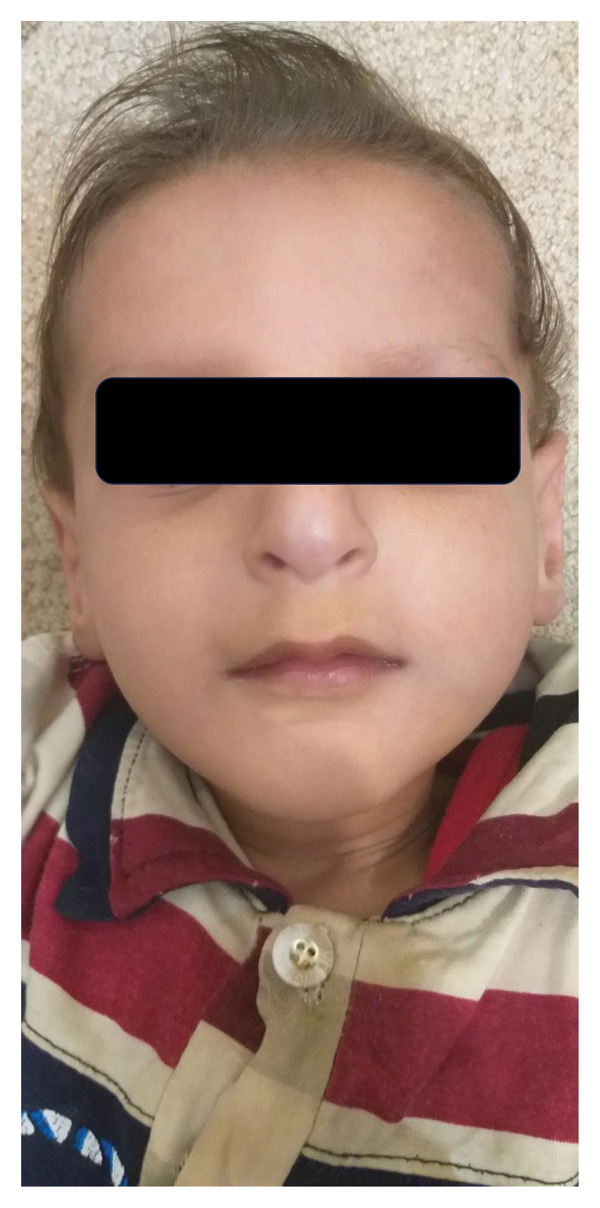
(b)

**Figure:**
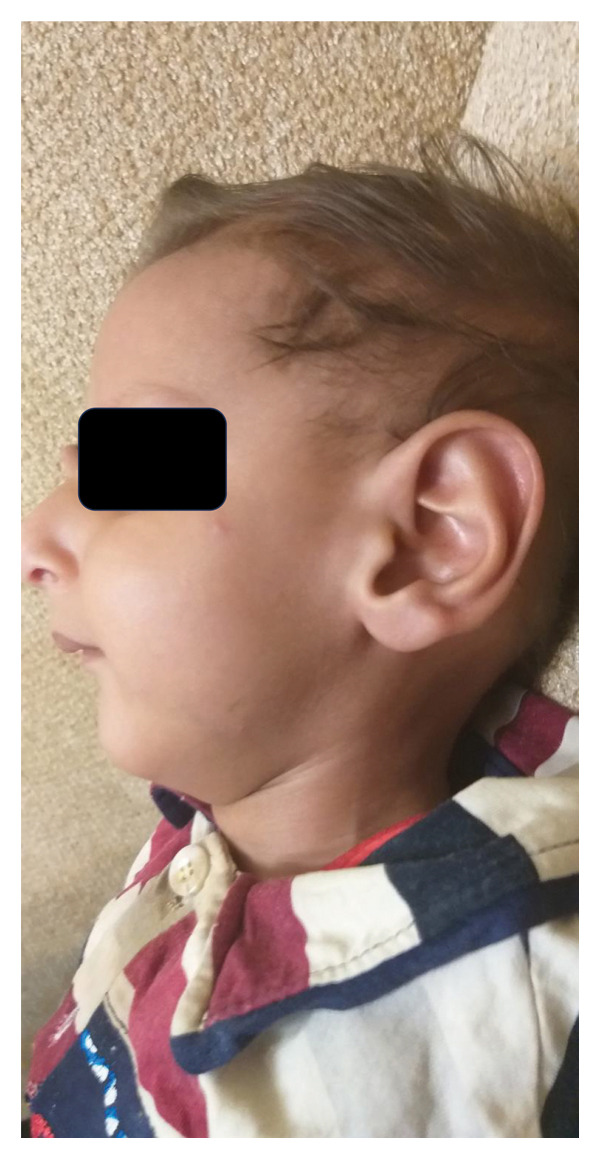
(c)

### 3.2. Molecular and Bioinformatics Analysis and Literature Review

Analysis of the WES data on subject V‐4 revealed a pathogenic nonsense variant c.C3862T, p.R1288X (NM_000124) in the *ERCC6* gene, which co‐segregated with the phenotype after Sanger sequencing (Figure 1(b)). The variant was submitted in ClinVar as pathogenic (VCV000031578.3). No other pathogenic exonic, intronic, synonymous, or compound heterozygous variants were identified in the exome file, which could be matched with the clinical presentation of our patients. The detected variant was predicted to be damaging by different pathogenicity predictive tools. The Phred‐scaled Combined Annotation Dependent Depletion score of this variant was 44. p.R1288X is assumed to cause loss of normal function of the encoded protein either through protein truncation or nonsense‐mediated mRNA decay. According to the evidence, this variant was classified as a pathogenic variant. Also, by reviewing the articles, the clinical information of nine other patients who are compound heterozygous or homozygous for this variant was collected (Table 1).

**Table 1:** Clinical summary of the present cases and previously reported cases with c.3862C>T deleterious variant.

*ERCC6* variant	c.3862C>T/c.3862C>T						c.3862C>T/c.3862C>T	c.3862C>T/c.2047C>T	c.3862C>T/c.2060C>T	c.3862C>T/c.3862C>T	
Clinical diagnosis	COFS						CS				
Family no.	1						2	3	4	Current study	
Patient no.	1	2	3	4	5	6	1	1	1	1	2
Ethnic region	Finland						Iran	UK	France	Iran	
Sex	M	F	F	M	M	M	F	F	M	M	M
Low birth weight	Yes	Yes	Yes	Yes	Yes	Yes	No	NA	Yes	Yes	Yes
Growth failure	Yes	Yes	Yes	Yes	Yes	Yes	Yes	NA	Yes	Yes	Yes
Age at onset	Congenital	Congenital	NA	NA	NA	NA	NA	NA	< 1	4 mo	3 mo
Age at death or latest evaluation	3 years, 7 mo	4 years, 2 mo	4 years, 6 mo	6 years, 1 mo	3 years, 9 mo	8 years, 5 mo	2	NA	3	3 years	6 mo
ID	Yes	Yes	Yes	Yes	Yes	Yes	Yes	NA	Yes	Yes	Yes
BHC	31 cm (−3.5 SD)	32 cm (−2.1 SD)	NA	NA	NA	NA	NA	NA	NA	33 cm	31 cm
Bird‐like face	Yes	Yes	Yes	Yes	NA	NA	Yes	Yes	Yes	Yes	NA
Microcephaly	Yes	Yes	Yes	Yes	Yes	Yes	Yes	NA	Yes	Yes	Yes
Developmental delay	Yes	Yes	Yes	Yes	Yes	Yes	NA	NA	NA	Yes	Yes
Sitting unsupported, standing, walking	Never	Never	Never	Never	Never	Never	NA	NA	NA	Never	Never
Abnormal speech	Yes	Yes	Yes	Yes	Yes	Yes	NA	NA	NA	Yes	Yes
Joint contracture	Yes	Yes	Yes	Yes	Yes	Yes	NA	NA	NA	Yes	NA
Muscle weakness	Yes	Yes	NA	NA	NA	NA	NA	NA	NA	Yes	Yes
Pulmonary insufficiency	Yes	Yes	NA	NA	NA	NA	NA	NA	NA	Yes	Yes
Feeding problem	Yes	Yes	NA	NA	NA	NA	NA	NA	NA	Yes	Yes
Eye contact	After cataract operation	After cataract operation	Follows light, not objects	Follows light, not objects	After cataract operation	After cataract operation	NA	NA	NA	Follows light and objects	Follows light and objects
Retinal degeneration	NA	NA	NA	NA	NA	NA	Yes	NA	Yes	NA	NA
Nystagmus	Yes	Yes	Yes	NA	No	Yes	NA	NA	NA	Yes	NA
Micrognathia	Yes	Yes	NA	NA	Yes	Yes	NA	NA	NA	No	No
Microphthalmia	Yes	Yes	Yes	Yes	Yes	Yes/No	NA	NA	Yes	Yes	NA
Sunken eyes	Yes	Yes	Yes	Yes	Yes	Yes	NA	NA	NA	Yes	NA
Congenital cataracts	Yes	Yes	NA	NA	Yes	Yes	NA	NA	Yes	No	No
Kyphosis/scoliosis	Yes	Yes	No	No	Yes	Yes	NA	NA	NA	Yes	NA
Flexion contractures	Yes	Yes	Yes	Yes	Yes	Yes	NA	NA	NA	Yes	NA
Arthrogryposis	Yes	NA	NA	NA	NA	NA	NA	NA	No	No	No
Seizures	No	Yes	No	No	No	Yes	NA	NA	NA	No	No
Elevated SGOT/SGPT	NA	NA	NA	NA	NA	NA	NA	NA	NA	Yes	NA
Deafness	Yes	Yes	NA	NA	NA	NA	Yes	NA	No	Yes	Yes
Clinical photosensitivity	Yes	No	NA	NA	NA	NA	Yes	NA	No	Yes	Yes
Skin dyspigmentation	No	No	No	No	No	No	NA	NA	NA	No	No
Anhidrosis	NA	NA	NA	NA	NA	NA	NA	NA	NA	Yes	Yes
Cold extremities	NA	Yes	NA	NA	NA	NA	NA	NA	NA	Yes	Yes
Thin lips	Yes	Yes	Yes	Yes	NA	NA	NA	NA	NA	No	No
Upper lip overlaps lower lip	Yes	Yes	Yes	Yes	Yes	Yes	NA	NA	NA	No	No
Large ear pinna	Yes	Yes	NA	NA	Yes	No	NA	NA	NA	Yes	Yes
Prominent nasal bridge	Yes	Yes	Yes	Yes	Yes	Yes	NA	NA	NA	Yes	NA
EEG	NA	Normal	NA	NA	NA	NA	NA	NA	NA	Normal	NA
MRI	Delayed myelination, hypoplasia of corpus callosum	Delayed myelination, hypoplasia of corpus callosum	NA	NA	NA	NA	NA	NA	NA	Brain atrophy, white matter abnormality. Thin corpus callosum	NA
Ref	[19]						[2]		[13]		

Abbreviations: BHC = birth head circumference, COFS = cerebrooculofacioskeletal syndrome, CS = Cockayne syndrome, ID = intellectual disability, mo = month, NA = not available, SGOT = serum glutamic oxaloacetic transaminase, and SGPT = serum glutamic‐pyruvic transaminase.

## 4. Discussion

In this study, WES was performed on a consanguineous Iranian family with two affected individuals. It revealed a pathogenic variant (NM_000124: c.3862C>T, p.R1288X) in the *ERCC6* gene that co‐segregated with the phenotype using Sanger sequencing (Figure 1). Interestingly, this variant has previously been reported in association with both CS and COFS [13, 16]. Clinical features of previously reported cases harboring the variant (NM_000124: c.3862C>T, p.R1288X) are summarized in Table 1.

Our patients’ manifestations were different from the reported cases with the same variant. Some symptoms of our patients are shared between both the CS and COFS including failure to thrive, photosensitivity, leukodystrophy, muscle contracture, intellectual disability, loss of subcutaneous fat, joint stiffness, hearing loss, and vision problems.

Furthermore, they showed some specific symptoms associated with both the CS and COFS. Although microcephaly in CS patients usually develops during the first 2 years of life, patients with COFS show congenital microcephaly [12, 18]. Both siblings had congenital microcephaly, as seen in COFS. The older sibling (V‐4) manifested some other COFS‐specific symptoms, such as severe developmental delay, micrognathia, and large ears; however, he did not exhibit arthrogryposis, cataracts, or microphthalmia, which are diagnostic criteria for COFS [19]. Likewise, patient V‐4 had increased eye discharge, particularly during sleep, which was an unusual finding given that in patients with *ERCC6* deleterious variants, the lacrimation decreases [7]. By and large, patient V‐4 showed an intermediate phenotype between CS and COFS. The younger affected brother (who was 6 months old at the time) did not exhibit some of the disorder’s symptoms, particularly in his facial features, because the phenotypic presentation of *ERCC6* gene deleterious variant develops over time and may not be recognized during the early stages of the disease [9, 20]. Furthermore, this variant has also been reported in a compound heterozygote state without detailed clinical manifestation (Table 1), so we could not compare their clinical symptoms with those of our patients. As mentioned, clinical symptoms in patients with a deleterious variant in the *ERCC6* gene may manifest with varying severity, ranging from a severe early‐onset (COFS) to a mild late‐onset form (CS type III) [21]. Different types of disease‐causing variants, such as missense, nonsense, frameshift, small deletions or insertions, large deletions, splicing, and a mid‐intronic variant in the *ERCC6* gene, have been reported [6, 13]. The researchers, by categorizing the CS into different subtypes based on the severity of the symptoms, attempted to find a clear‐cut genotype–phenotype correlation but were unsuccessful [2, 3]. The presence of the same variant in several patients with different phenotypes provides additional evidence that COFS is a severe form of CS and they are existing on a continuum. Moreover, this study’s findings indicate that probably unknown epigenetic, environmental factors or modifier genes contribute significantly to the severity of CS symptoms. Modifier gene is traditionally defined as “a gene that affects the phenotypic expression of another gene” which affects many human traits [22]. Modifier genes have the ability to influence the penetrance, dominance, expressivity, and pleiotropy of a phenotype [23]. Despite the challenge in identifying modifier genes, identifying these genes may be of great interest from the viewpoints of treatment, course of the disease, and genetic counseling [22]. In light of this, we invite researchers to do more investigation to identify CS‐modifier genes, which can not only help researchers find exact genotype–phenotype correlations but also help to predict disease prognosis.

In conclusion, the findings of this study expand the clinical spectrum of *ERCC6* variants and provide more supporting evidence in order to confirm that CS and COFS are phenotypic spectrums rather than different conditions. It also shows that genetic and epigenetic factors probably play a pivotal role in the severity of symptoms. Additional patients and the accumulation of more clinical data are needed to identify the modifier genes as well as to find a clear‐cut genotype–phenotype correlation.

## Ethics Statement

This study was approved by the Ethical Committee of Isfahan University of Medical Sciences (IR.MUI.MED.REC.1398.186).

## Consent

Written informed consent was obtained from all participants or their legal guardians.

## Disclosure

All authors have read and approved the final manuscript to be published and agreed to be responsible for the accuracy of the data and details.

## Conflicts of Interest

The authors declare no conflicts of interest.

## Author Contributions

Mehdi Khorram: contributed in data analysis and writing the draft. Erfan Khorram contributed in investigation, visualization, and writing the draft. Mohammad Amin Tabatabaiefar and Omid Yaghini contributed in investigation and data collection. Omid Iravani contributed in data analysis and supervised the clinical evaluation. Vida Yazdani, Mitra Pakbaz, and Aida Kheirollahi contributed in data collection and laboratory works. Majid Kheirollahi designed and supervised the study.

## Funding

This work was supported by Isfahan University of Medical Sciences (grant number: 394950).

## Data Availability

Data supporting the findings of this study are available upon request from the corresponding author.
